# Perioperative Complications are Associated With Adverse Long-Term Prognosis and Affect the Cause of Death After General Surgery

**DOI:** 10.1007/s00268-016-3600-4

**Published:** 2016-06-14

**Authors:** Elke K.M. Tjeertes, K. H. J. Ultee, R. J. Stolker, H. J. M. Verhagen, F. M. Bastos Gonçalves, A. G. M. Hoofwijk, S. E. Hoeks

**Affiliations:** 1 Department of Anesthesiology, Erasmus University Medical Centre, Room H-1273, PO Box 2040 3000, CA Rotterdam, The Netherlands; 2 Department of Vascular Surgery, Erasmus University Medical Centre, Rotterdam, The Netherlands; 3 Department of Vascular Surgery, Hospital de Santa Marta, Lisbon, Portugal; 4 Department of Surgery, Zuyderland Medical Centre, Sittard, The Netherlands

## Abstract

**Background:**

It is unclear how mortality and causes of death vary between patients and surgical procedures and how occurrence of postoperative complications is associated with prognosis. This study describes long-term mortality rates and causes of death in a general surgical population. Furthermore, we explore the effect of postoperative complications on mortality.

**Methods:**

A single-centre analysis of postoperative complications, with mortality as primary endpoint, was conducted in 4479 patients undergoing surgery. We applied univariate and multivariable regression models to analyse the effect of risk factors, including surgical risk and postoperative complications, on mortality. Causes of death were also explored.

**Results:**

75 patients (1.7 %) died within 30 days after surgery and 730 patients (16.3 %) died during a median follow-up of 6.3 years (IQR 5.8–6.8). Significant differences in long-term mortality were observed with worst outcome for patients undergoing high-risk vascular surgery (HR 1.5; 95 % CI 1.2–1.9). When looking at causes of death, high-risk surgery was associated with a twofold higher risk of cardiovascular death (HR 1.9; 95 % CI 1.2–3.1), whereas the intermediate-risk group had a higher risk of dying from cancer-related causes (HR 1.5; 95 % CI 1.1–2.0). Occurrence of complications—particularly of cardiovascular nature— was associated with worse survival (HR 1.9; 95 % CI 1.3–2.7).

**Conclusion:**

High-risk vascular surgery and occurrence of postoperative complications are important predictors of late mortality. Further focus on these groups of patients can contribute to reduced morbidity. Improvement in quality of care should be aimed at preventing postoperative complications and thus a better outcome in a general surgical population.

## Introduction

Each year, more than 230 million major surgical procedures are performed worldwide [1]. Risk of mortality after surgery differs greatly in patients and surgical procedures. Also, evidence increasingly suggests that patients experiencing postoperative complications have a reduced quality of life and life expectancy itself [2–4]. Khuri et al. demonstrated that this adverse effect of complications on late mortality is independent of patients’ preoperative risk factors [2]. However, it is unknown if the causes of death are also effected. Recently, a large cohort study described important variations in postoperative mortality rates between European nations [5]. Both findings highlight room for improvement of perioperative care. In order to adequately inform patients of significant surgery risks, information on surgery-related complications and mortality is important.

The objective of our study is to describe long-term mortality rates and cause of death in a general surgical population. Furthermore, in addition to demographic and disease specific factors, we explored the effect of postoperative complications on long-term mortality.

## Methods

### Study sample

This study was performed in the Zuyderland medical centre, a medium-sized regional hospital in the Netherlands. This hospital contains a modern degree of automation and a reliable registration of the electronic medical record. A prospective database is used containing data on all surgical procedures performed [6]. The study complies with the Helsinki statement on research ethics, and the local medical ethical committee gave formal review and approval. Data were collected from patients who underwent elective or urgent non-cardiac surgery. We identified 5373 consecutive patients undergoing surgery from March 2005 to December 2006. Patients younger than 14 years old and patients undergoing surgical procedures under local infiltration were excluded. Because one of our primary endpoints was long-term survival, a patient’s first operation within the enrollment period was considered the index operation, and survival was determined from that moment onward. However, when a patient needed repeated surgery during the same hospital stay, we included the need for a re-operation as a separate outcome measure. A total of 4479 patients were considered suitable for the final study population.

### Baseline characteristics

Individual data on the patient’s medical history were obtained by a surgeon or a surgical resident prior to surgery. Data collected included main diagnosis, history of cardiac, pulmonary or cerebrovascular disease, diabetes mellitus, hypertension, ASA classification [7], any malignancy, as well as intoxications, use of medication, and patient’s height and bodyweight. Information from the electronic medical record on baseline characteristics could be completed in 96 up to 100 %, except for information on smoking habits, which could be obtained in 75 % of patients.

Pathological cardiac history was defined as a condition involving coronary artery disease, heart failure, valvular heart disease, arrhythmias or cardiomyopathy. Pulmonary disease was defined as illness of the lungs or respiratory system, such as COPD, asthma, lung cancer, chronic infections or previous embolisms. A previous cerebral thrombosis, embolism or hemorrhage was noted as cerebrovascular disease. Table 1 shows the surgical procedures, classified according to the standardized Dutch classification system [8]. For the purpose of this study, we categorized the main surgical procedures into fifteen generally accepted groups, which were then distributed over three risk categories: low-, intermediate- and high-risk procedures [9]. In this general teaching hospital, trauma patients are physiologically stable and patients undergoing highly complex low-volume surgery are being treated in tertiary university hospitals. Information on whether the patient had surgery requiring hospitalization or day-case surgery was also collected. Finally, we documented the type of anaesthesia, divided into general and/or regional. Validation of the database using a random sampling audit procedure confirmed a high level of accuracy and completeness of data.

**Table 1:** Surgical Categories according to the standardized Dutch classification system^8^

Surgical Categories	
Low-risk surgery	
	Breast surgery
	Hernia surgery^a^
	Minor surgery of soft tissue
	Minor trauma surgery
	Perianal surgery
	Varicose vein surgery
Intermediate-risk surgery	
	Appendectomy
	Carotid artery surgery
	Cholecystectomy
	Head and neck surgery
	Major abdominal surgery^b^
	Major trauma surgery^c^
	Thoracic surgery
High-risk surgery	
	Ischemic limb amputation
	Major vascular surgery^d^

^a^Except for incisional hernia repair

^b^I.e. liver, gastric, bowel, spleen, oesophagus and incisional hernia surgery

^c^I.e. multi trauma or trauma involving the femur or the hip

^d^I.e. open aortic repair and peripheral bypass surgery

### Outcome

All-cause mortality was the primary endpoint of this study. Secondary endpoints were postoperative complications within 30 days after surgery and cause of death. For the evaluation of outcome, a surgical resident followed patients during hospitalization and postoperative visits to the outpatient clinic up to 1 year. We gathered the following data: date of surgery, date of discharge, length of hospital stay, operating time, blood loss and postoperative complications. Complications were defined as any event deviating from a normal postoperative course within 30 days after surgery. We separately documented the following postoperative complications: wound infections, pneumonia, cardiovascular and cerebrovascular events, deep vein thrombosis and/or pulmonary embolisms, ICU-admission, readmission and need for complication surgery. A surgical resident as well as a member of the surgical staff independently scored complications. For an objective interpretation of outcome, we used the earlier proposed Clavien–Dindo classification system as guidance [10]. Complications were subsequently divided into 4 categories: no complication, a self-limiting complication (for example, a small wound dehiscence not needing specific treatment), a non-self-limiting complication (for example, the need for antibiotics in case of pneumonia or wound infection, a re-operation, or a CT-guided drainage of an abscess) and a major complication, which involves complications with residual disability, including organ failure.

Information on long-term mortality and cause of death were obtained by inquiry of the national public register and Dutch Central Bureau of Statistics. Autopsy was not routinely performed, and the expected cause leading to health deterioration prior to death was considered the underlying cause of death, in parallel to the strategy used for the overall Dutch population. The causes of death were grouped according to the International Classification of Diseases, 10th Revision (ICD-10). For patients who lived abroad, last available follow-up information was used. For better understanding of surgical outcome, we compared our study population with a general age and gender matched Dutch population. Information about the general population was extracted from the Electronic Databank of Central Bureau of Statistics Netherlands [11].

### Statistical analysis

Categorical variables are presented as numbers and percentages. Continuous data are presented as mean ± standard deviation (SD) when normally distributed or as median values and corresponding 25^th^ and 75^th^ percentiles when data were skewed. We used Chi-square test for comparison of categorical variables and analysis of variance or Kruskal–Wallis test for continuous variables. Univariate and multivariable Cox regression models were used to evaluate association between surgical risk categories and mortality. Low-risk surgery was used as reference category in the regression analyses. To ensure we give a true estimate of mortality risk, we entered all potential confounders (age, gender, type of anaesthesia, ASA classification, diabetes, hypertension, pulmonary- , cardiac- or cerebrovascular disease, BMI, malignancy) in the multivariable regression models.

Kaplan–Meier survival curves were calculated for each type of surgical category. The predictive value of postoperative complications on cause-specific long-term mortality was assessed in 30-day survivors using Cox regression analysis.

Since it seems predictable that patients undergoing high-risk procedures are more at risk of experiencing postoperative complications and death, we performed an additional sensitivity analysis, excluding this high-risk surgery group.

Results are reported as hazard ratios (HR) with a 95 % confidence interval. Significance was set at a two-sided *P* value <0.05. Analysis was performed using SPSS software version 20.0.0.

## Results

### Patient population

4479 patients undergoing general surgery were included in this analysis. There were an equal percentage of men and women in the cohort and mean age was 55.0 ± 17.5 years. Table 2 shows clinical baseline and surgery-related characteristics of the study population. The majority of procedures (85.6 %) were performed under general anaesthesia. Most of the procedures (56.4 %) could be classified as low-risk surgery according to the surgical risk classification system [9]. Intermediate- and high-risk surgery accounted for 38.4 and 5.2 %, respectively.

**Table 2:** Baseline characteristics

	All patients (N = 4479)	Any complication (N = 949)	Overall mortality (N = 730)
Demographics			
Age, years (mean ± SD)	55.0 ± 17.5	62.7 ± 16.8^#^	71.6 ± 12.3^##^
Male sex (%)	2307 (51.5 %)	495 (52.2 %)	402 (55.1 %)^##^
ASA classification (%)		^#^	^##^
I	1501 (33.5 %)	157 (16.6 %)	19 (2.6 %)
II	1600 (35.7 %)	292 (31.0 %)	149 (20.6 %)
III	1169 (26.1 %)	405 (42.9 %)	428 (59.0 %)
IV	161 (3.6 %)	85 (9.0 %)	125 (17.2 %)
V	4 (0.1 %)	4 (0.4 %)	4 (0.6 %)
Medical history (%)			
Diabetes mellitus	402 (9.1 %)	142 (15.1 %)^#^	160 (22.2 %)^##^
Hypertension	884 (20.0 %)	261 (27.8 %)^#^	259 (35.9 %)^##^
Cerebrovascular disease	313 (7.1 %)	92 (9.8 %)^#^	132 (18.3 %)^##^
Malignant disease	1028 (23.2 %)	296 (31.4 %)^#^	358 (49.4 %)^##^
Pathological cardiac history	825 (18.6 %)	294 (31.3 %)^#^	358 (49.7 %)^##^
Pathological pulmonary history	633 (14.3 %)	186 (19.8 %)^#^	221 (30.7 %)^##^
Smoking* (%)		^#^	^##^
Current smoking	1075 (32.1 %)	202 (29.2 %)	183 (32.8 %)
History	590 (17.6 %)	143 (20.7 %)	157 (28.1 %)
No smoking	1682 (50.3 %)	346 (50.1 %)	218 (39.1 %)
BMI category (%)		^#^	^##^
Normal weight (BMI 18,5-25 kg/m2)	1815 (42.3 %)	339 (37.8 %)	331 (48.2 %)
Underweight (BMI < 18,5 kg/m2)	100 (2.3 %)	28 (3.1 %)	35 (5.1 %)
Overweight (BMI 25-30 kg/m2)	1635 (38.1 %)	345 (38.5 %)	212 (30.9 %)
Obese (BMI > 30 kg/m2)	743 (16.6 %)	185 (20.6 %)	109 (15.9 %)
Surgical categories (%)		^#^	^##^
Low-risk surgery	2527 (56.4 %)	302 (31.8 %)	238 (32.6 %)
Breast	382 (8.5 %)	49 (5.2 %)	63 (8.6 %)
Hernia	839 (18.7 %)	88 (9.3 %)	79 (10.8 %)
Minor surgery of soft tissue	408 (9.1 %)	66 (7.0 %)	58 (7.9 %)
Minor trauma	228 (5.1 %)	27 (2.8 %)	12 (1.6 %)
Perianal surgery	278 (6.2 %)	19 (2.0 %)	14 (1.9 %)
Varicose vein surgery	392 (8.8 %)	53 (5.6 %)	12 (1.6 %)
Intermediate-risk surgery	1720 (38.4 %)	534 (56.3 %)	367 (50.3 %)
Appendectomy	251 (5.6 %)	55 (5.8 %)	11 (1.5 %)
Carotid artery	74 (1.7 %)	12 (1.3 %)	15 (2.1 %)
Cholecystectomy	495 (11.1 %)	100 (10.5 %)	30 (4.1 %)
Head and neck	102 (2.3 %)	30 (3.2 %)	8 (1.1 %)
Major abdominal	629 (14.0 %)	295 (31.1 %)	222 (30.4 %)
Major trauma	79 (1.8 %)	27 (2.8 %)	46 (6.3 %)
Thoracic	90 (2.0 %)	15 (1.6 %)	35 (4.8 %)
High risk surgery	232 (5.2 %)	113 (11.9 %)	125 (17.1 %)
Amputation	36 (0.8 %)	18 (1.9 %)	29 (4.0 %)
Major vascular	196 (4.4 %)	95 (10.0 %)	96 (13.2 %)
Surgery characteristics (%)			
General anaesthesia	3824 (85.6 %)	866 (91.7 %)^#^	654 (90.0 %)^##^
Outpatient surgery	1539 (34.4 %)	139 (14.6 %)^#^	67 (9.2 %)^##^
Length of stay, (days) (median + IQR)	2 (1–8)	8 (2–16)	7 (2–15)
Blood Loss, (mL) (median + IQR)	15 (5–50)	50 (20–250)	50 (10–200)
Operation time, (min) (median + IQR)	41 (25–68)	63 (38–110)	61 (35–113)

^#^ Significantly different (p <05) when compared to patients without complications

^## ^Significantly different (p <05) when compared to alive patients

* Data available in 75.7 % of patients

Table 3 shows baseline characteristics according to the main surgical categories. As expected, demographics and proportion of comorbidities varied widely when categorized by different surgical procedures. Patients with trauma of the hip and major vascular patients were of higher age (76.1 ± 17.2 and 69.8 ± 10.7, respectively). In general, patients who underwent vascular surgery had the highest prevalence of comorbid diseases.

**Table 3:** Baseline characteristics according to main surgical categories

	Male (%)	Age, years Mean ± SD	HT (%)	DM (%)	Cerebrovascular disease (%)	Malignant disease (%)	Pathological cardiac history (%)	Pathological pulmonary history (%)	Current smoking (%)
Low surgical risk	1408 (55.7 %)	52.2 (± 16.4)	380 (15.2 %)	152 (6.1 %)	94 (3.8 %)	511 (20.5 %)	339 (13.6 %)	287 (11.5 %)	604 (32.6 %)
Breast	19 (5.0 %)	57.3 (± 14.8)	80 (20.9 %)	33 (8.6 %)	14 (3.7 %)	333 (87.2 %)	52 (13.6 %)	41 (10.7 %)	91 (28.2 %)
Hernia	739 (88.1 %)	56.5 (± 15.6)	126 (15.1 %)	47 (5.6 %)	45 (5.4 %)	48 (5.8 %)	152 (18.2 %)	96 (11.5 %)	194 (30.1 %)
Minor surgery of soft tissue	226 (55.4 %)	49.8 (± 16.5)	77 (18.9 %)	37 (9.1 %)	17 (4.2 %)	97 (23.8 %)	59 (14.5 %)	66 (16.2 %)	102 (34.8 %)
Minor trauma	102 (44.7 %)	48.3 (± 18.5)	34 (16.3 %)	13 (6.2 %)	7 (3.3 %)	7 (3.3 %)	21 (10.0 %)	20 (9.6 %)	45 (32.8 %)
Perianal surgery	207 (74.5 %)	40.5 (± 15.7)	21 (7.8 %)	14 (5.2 %)	5 (1.9 %)	9 (3.3 %)	28 (10.4 %)	21 (7.8 %)	98 (50.0 %)
Varicose vein surgery	115 (29.3 %)	50.9 (± 13.0)	42 (10.7 %)	8 (2.0 %)	6 (1.5 %)	17 (4.3 %)	27 (6.9 %)	43 (11.0 %)	74 (28.6 %)
Intermediate surgical risk	739 (43.0 %)	57.0 (± 18.4)	394 (23.1 %)	181 (10.6 %)	174 (10.2 %)	489 (28.6 %)	347 (20.3 %)	288 (16.9 %)	388 (29.6 %)
Appendectomy	126 (50.2 %)	39.6 (± 17.9)	24 (9.7 %)	8 (3.2 %)	4 (1.6 %)	12 (4.9 %)	14 (5.7 %)	8 (3.2 %)	51 (27.7 %)
Carotid artery	53 (71.6 %)	67.4 (± 9.3)	31 (41.9 %)	18 (24.3 %)	60 (81.1 %)	7 (9.5 %)	32 (43.2 %)	10 (13.5 %)	28 (41.2 %)
Cholecystectomy	152 (30.7 %)	54.0 (± 15.2)	97 (19.6 %)	38 (7.7 %)	24 (4.9 %)	36 (7.3 %)	74 (15.0 %)	48 (9.7 %)	123 (31.9 %)
Head and neck	41 (40.2 %)	54.1 (± 15.0)	20 (19.8 %)	9 (8.9 %)	4 (4.0 %)	22 (21.8 %)	7 (6.9 %)	8 (7.8 %)	23 (34.3 %)
Major abdominal	292 (46.4 %)	62.8 (± 16.7)	175 (28.0 %)	84 (13.4 %)	59 (9.4 %)	340 (54.2 %)	154 (24.6 %)	110 (17.6 %)	122 (25.8 %)
Major trauma	22 (27.8 %)	76.1 (± 17.2)	27 (35.5 %)	19 (25.0 %)	14 (18.4 %)	16 (20.8 %)	38 (50.0 %)	23 (30.3)	9 (18.0 %)
Thoracic	53 (58.9 %)	60.0 (± 15.7)	20 (22.2 %)	5 (5.6 %)	9 (10.0 %)	56 (62.2 %)	28 (31.1 %)	81 (90.0 %)	32 (38.1 %)
High surgical risk	160 (69.0 %)	69.7 (± 11.5)	110 (47.8 %)	69 (30.0 %)	45 (19.6 %)	28 (12.2 %)	139 (60.4 %)	58 (25.2 %)	83 (45.4 %)
Amputation	18 (50.0 %)	68.8 (± 15.2)	16 (44.4 %)	19 (52.8 %)	13 (36.1 %)	4 (11.1 %)	21 (58.3 %)	9 (25.0 %)	11 (40.7 %)
Major vascular	142 (72.4 %)	69.8 (± 10.7)	94 (48.5 %)	50 (25.8 %)	32 (16.5 %)	24 (12.4 %)	118 (60.8 %)	49 (25.3 %)	72 (46.2 %)

### Postoperative complications

We evaluated the effect of different surgical categories on postoperative outcome (Table 4). Complications occurred in 949 patients (21.0 %). In general, patients who experienced complications were of higher age (62.7 ± 16.8) when compared to all patients and had more comorbidities (Table 2). Amputation of an ischemic limb and major vascular surgery was associated with highest risk of complications (50.0 and 48.5 %). As expected, non-self-limiting complications (32.8 %), major complications (4.7 %) and 30-day mortality (8.6 %) were more often seen in the high-risk group.

**Table 4:** Postoperative Outcome within 30 Days

	No complication (%)	Self-limiting complication (Grade 1) (%)	Non-self-limiting complication (Grade 2 + 3) (%)	Major complication (Grade 4) (%)	Death (Grade 5) (%)
Low-risk surgery	2225 (88.0)	77 (3.0)	216 (8.5)	1 (0.0)	8 (0.3)
Breast	333 (87.2)	13 (3.4)	36 (9.4)	0 (0.0)	0 (0.0)
Hernia	751 (89.5)	19 (2.3)	65 (7.7)	1 (0.1)	3 (0.4)
Minor surgery of soft tissue	342 (83.8)	18 (4.4)	44 (10.8)	0 (0.0)	4 (1.0)
Minor trauma	201 (88.2)	8 (3.5)	19 (8.3)	0 (0.0)	0 (0.0)
Perianal surgery	259 (93.2)	5 (1.8)	13 (4.7)	0 (0.0)	1 (0.4)
Varicose vein surgery	339 (86.5)	14 (3.6)	39 (9.9)	0 (0.0)	0 (0.0)
Intermediate-risk surgery	1186 (69.0)	99 (5.8)	349 (20.3)	39 (2.3)	47 (2.7)
Appendectomy	196 (78.1)	16 (6.4)	37 (14.7)	1 (0.4)	1 (0.4)
Carotid artery	62 (83.8)	5 (6.8)	2 (2.7)	3 (4.1)	2 (2.7)
Cholecystectomy	395 (79.8)	30 (6.1)	64 (12.9)	3 (0.6)	3 (0.6)
Head and neck	72 (70.6)	11 (10.8)	19 (18.6)	0 (0.0)	0 (0.0)
Major abdominal	334 (53.1)	31 (4.9)	198 (31.5)	29 (4.6)	37 (5.9)
Major trauma	52 (65.8)	2 (2.5)	19 (24.1)	3 (3.8)	3 (3.8)
Thoracic	75 (83.3)	4 (4.4)	10 (11.1)	0 (0.0)	1 (1.0)
High risk surgery	119 (51.3)	6 (2.6)	76 (32.8)	11 (4.7)	20 (8.6)
Amputation	18 (50.0)	2 (5.6)	9 (25.0)	1 (2.8)	6 (16.7)
Major vascular	101 (51.5)	4 (2.0)	67 (34.2)	10 (5.1)	14 (7.1)
All types	3530 (78.8)	182 (4.1)	641 (14.3)	51 (1.1)	75 (1.7)

### All-cause mortality

**Fig. 1:**
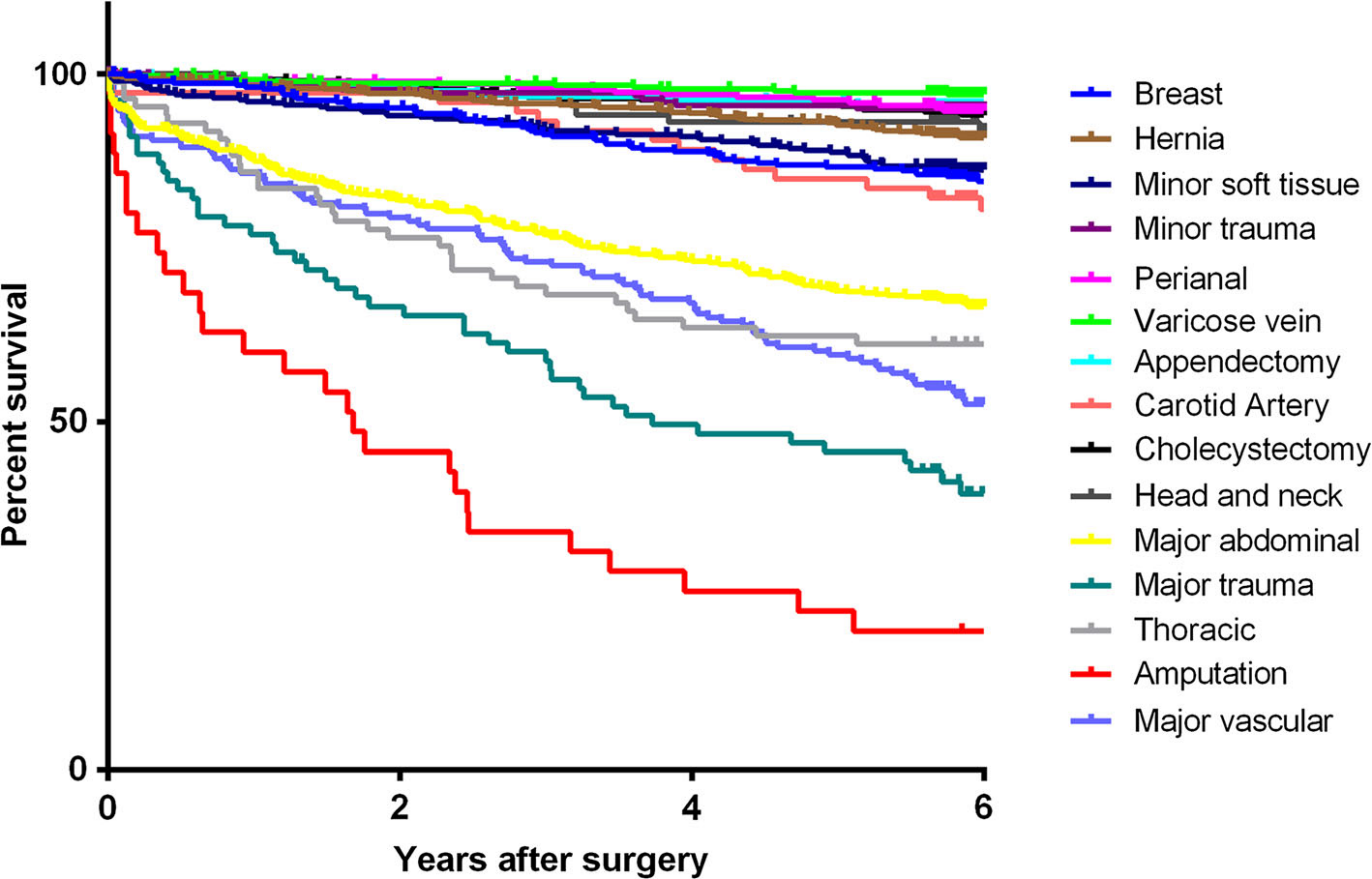
Kaplan–Meier estimates of long-term survival among different surgical procedures

**Fig. 2:**
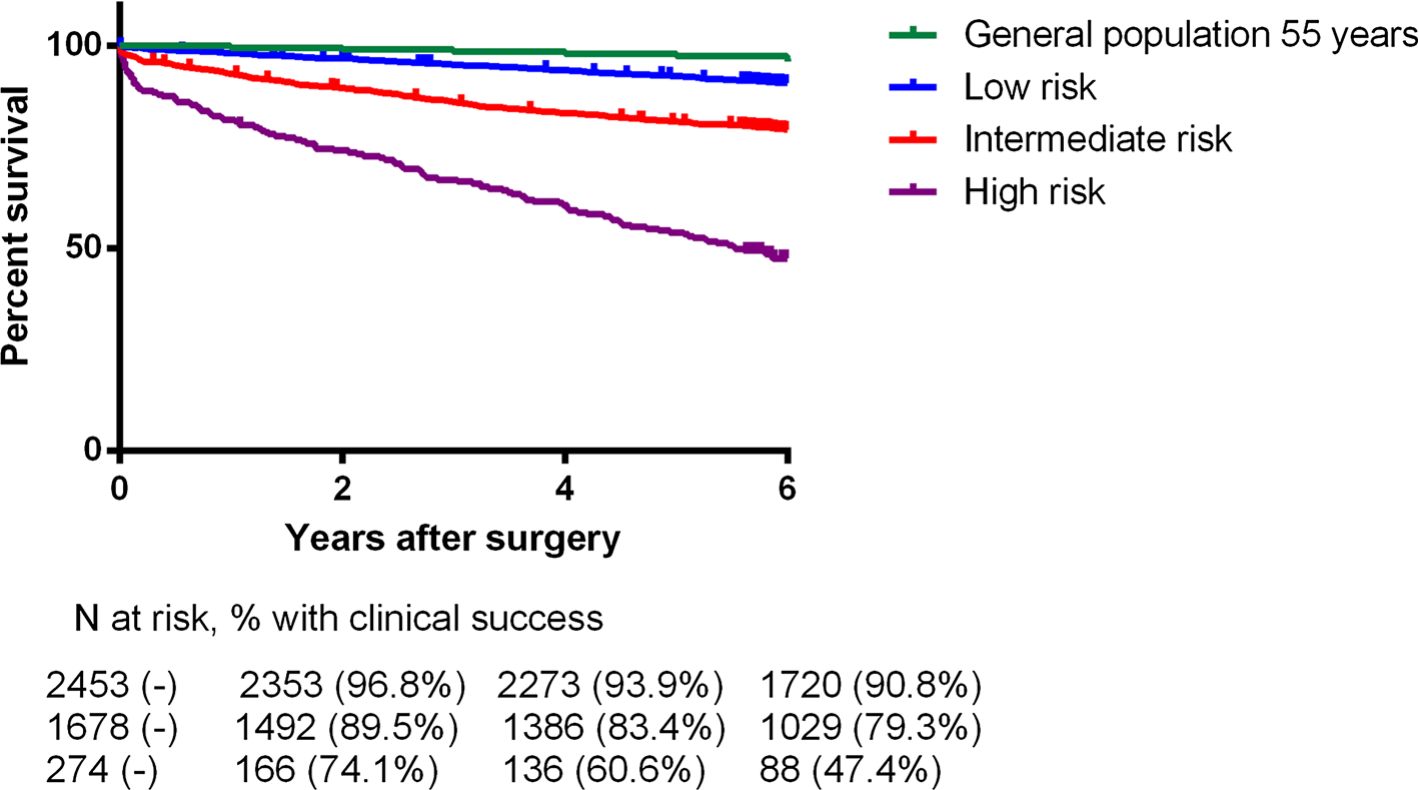
Kaplan–Meier estimates of long-term survival among different surgical categories, including a survival curve of the age and gender matched general Dutch population

Overall 30-day mortality rate was 1.7 % (75 patients), with cardiac and cancer-related death accounting for 26.6 and 19.0 %, respectively. Information on long-term mortality and cause of death was available in 96.4 % of patients. For patients who lived abroad or had immigrated (*N* = 108, 2.4 %), last available follow-up information was used. The primary endpoint of all-cause mortality was observed in 730 patients (16.3 %) during median follow-up of 6.3 years (IQR 5.8–6.8). When comparing risk of mortality associated with types of surgery, confounding factors such as demographics and comorbidities must be taken into account. Table 5 shows important differences in long-term mortality in relation to surgical risk in a multivariable regression model. Patients who underwent intermediate (HR 1.2; 95 % CI 1.0–1.5) or high-risk surgery (HR 1.5; 95 % CI 1.2–1.9) had a significant higher relative mortality risk. Figures 1 and 2 show Kaplan–Meier estimates of long-term survival among different surgical procedures and categories. In order to interpret the effect of surgery on long-term survival, Fig. 2 also shows the survival curve of the age and gender matched general Dutch population.

**Table 5:** The association between surgery risks and different mortality hazards

	Events	Univariate	Multivariable*		
	N (%)	Hazard Ratio	95 % CI	Hazard Ratio	95 % CI
Overall mortality					
Low-risk surgery	238	1	–	1	–
Intermediate-risk surgery	367	2.364	1.998–2.796	1.216	1.017–1.455
High-risk surgery	125	7.512	6.014–9.382	1.507	1.166–1.946
Cardiovascular mortality					
Low-risk surgery	57	1	–	1	–
Intermediate-risk surgery	58	1.686	1.170–2.431	0.860	0.574–1.287
High-risk surgery	46	12.747	8.621–18.848	1.923	1.194–3.095
Cancer-related mortality					
Low-risk surgery	93	1	–	1	–
Intermediate-risk surgery	192	3.301	2.574–4.233	1.503	1.143–1.977
High-risk surgery	21	3.615	2.270–5.758	1.281	0.762–2.152

^a^Analyses were adjusted for age, gender, type of anaesthesia, ASA classification, diabetes, hypertension, pulmonary-, cardiac- or cerebrovascular disease, BMI and the presence of a malignancy

### Late causes of death

When looking at the cause of late mortality, patients in the high-risk group, i.e., vascular patients, had a twofold higher risk of cardiovascular death (HR 1.9; 95 % CI 1.2–3.1) compared to low-risk patients (Table 5). Patients in the intermediate group, i.e., the group consisting of most patients undergoing cancer surgery (28.6 %), had a higher risk of dying from a cancer-related cause (HR 1.5; 95 % CI 1.1–2.0).

### Association between postoperative complications and mortality

**Fig. 3:**
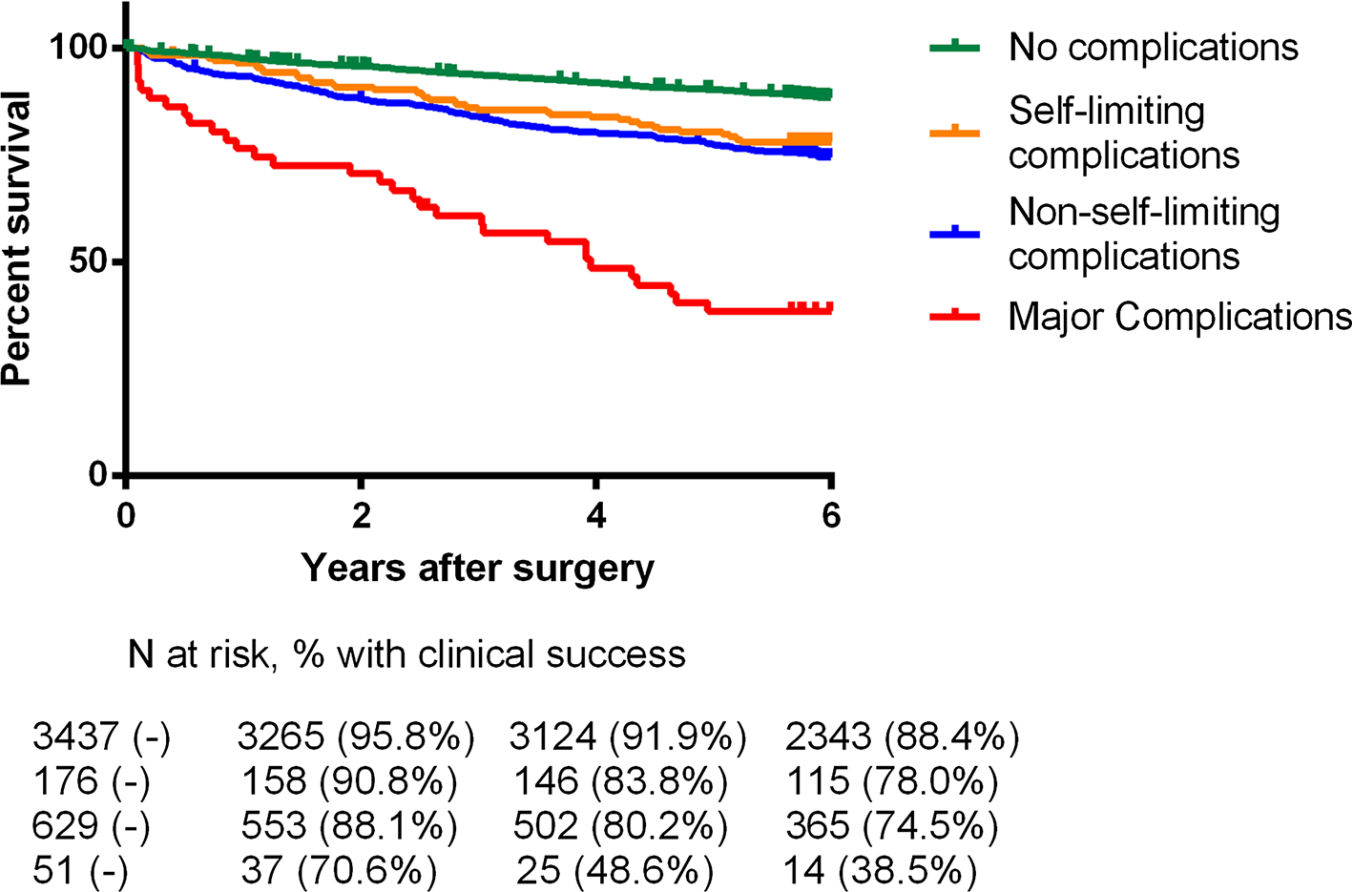
Kaplan–Meier estimates of 30-day survivors, calculated for different types of complications

We found a significant adverse effect between the presence of postoperative complications and long-term mortality (Table 6). Figure 3 shows a Kaplan–Meier estimate of 30-day survivors, calculated for different types of complications. This survival curve illustrates that survival in the patient group with self-limiting complications is already worse compared to those with no complications, whereas the prognosis in the two patient groups with non-self-limiting and major complications is considerably and increasingly worse. After exclusion of high-risk procedures, this association between complications and mortality still remained significant in low- and intermediate-surgical risk patients (HR 1.2; 95 % CI 1.1–1.5).

**Table 6:** The association between 30-day complications and different long-term mortality hazards (in 30-day survivors)

	Events	Univariate	Multivariable		
	N (%)	Hazard Ratio	95 % CI	Hazard Ratio	95 % CI
Overall mortality	627	2.393	2.033–2.818	1.197	1.009–1.421
Cardiovascular mortality	140	3.527	2.526–4.924	1.890	1.312–2.721
Cancer-related mortality	291	2.230	1.748–2.845	1.101	0.850–1.426

^a^Analyses were adjusted for age, gender, type of anaesthesia, ASA classification, diabetes, hypertension, pulmonary-, cardiac- or cerebrovascular disease, BMI and the presence of a malignancy

## Discussion

Late mortality after surgery might be higher than expected (16 % at 6 years). The 30-day mortality of 1.7 % we found is much similar to the 1.9 % reported in a study performed in the Netherlands in 2010 [12]. Previous studies on outcome following surgery are scarce, mostly retrospective in design and based on administrative databases [12–14]. Lee et al. demonstrated that clinical chart review had a significantly better accuracy than a comparable administrative database model, probably due to undercoding of comorbidities in the latter [15].

Instead of focussing on demographic and disease specific factors only, we took variables such as postoperative complications into account, which have been reported to be of clinical importance [2, 3, 16, 17]. To our knowledge, this is the first prospective study to combine all these factors in order to analyse long-term outcome, including cause of late death.

According to this study, special focus on two groups of patients is advised in order to improve postoperative care. As can be expected, the first group associated with an adverse outcome is the group of high-risk vascular patients. Although these were only 5 % of operated patients, they accounted for 27 % of 30-day mortality. The high incidence of postoperative death in this subpopulation is in accordance with previous literature [9, 12, 18]. Taking the surgical procedure itself into account when predicting risk of postoperative complications, rather than patients’ comorbidity only, remains very important.

The second group highly and independently associated with late death has patients who experience postoperative complications. In this study, complications occurred in 21 % of patients, who were mostly of higher age and had more comorbidities. In order to better understand, the relationship between postoperative complications and reduced survival one might ask if a complication is the cause of this reduction, or a sign of a bigger pathological problem. In this study, we demonstrated that the relationship between complications and reduced survival remains valid even after adjusting for potential confounders. Moreover, after exclusion of high-risk surgery in a sensitivity analysis, this relationship still remains significant.

Recent literature shows that frailty is associated with higher morbidity and mortality, independent of other risk factors in a surgical population. Preoperative recognition of this multidimensional vulnerability may be an adjunct in assessment of preoperative risk factors. Also, evidence has shown that the surgical procedure itself elicits a stress response, initiated by tissue injury [19–22]. Surgical injury profoundly affects the innate and adaptive immune responses, leading to an increased susceptibility to complications [22].

Cause-specific mortality analysis showed that the high-risk group had a twofold higher risk of dying from a cardiovascular cause. As expected, patients in the intermediate surgery group were more likely to die from a cancer-related cause. In order to appreciate these numbers, the Dutch registration for cause of death needs clarification. The certificate of death filled in by a medical practitioner is based on guidelines of the World Health Organization [23]. Only one cause can be coded as primary cause of death. Primary cause of death is the cause of the initial health deterioration leading to the end of life. For example, if a patient had surgery because of intestinal cancer and died due to postoperative myocardial infarction, cause of death would be cancer-related and the myocardial event noted as a secondary response to his underlying illness [24]. Reliability of cause-of-death coding in the Netherlands turns out to be high (>90 %) for major causes of death, such as cancer- and cardiovascular-related causes [24, 25].

We recognize that our study has potential limitations. It is conducted in a single centre with a potential bias in referral pattern. However, at the time of enrolment, only patients needing total pelvic exenteration or patients with severe comorbidity were referred to tertiary centres. All other major abdominal surgery, such as liver, gastric, bowel, spleen, oesophagus and incisional hernia surgery, was performed in this regional hospital. Our data contain quite a large number of intermediate- and high-risk procedures and procedures with a long operation time.

Second, we only included patients who underwent surgery. There might have been high-risk patients screened for surgery, but denied because of the risk of potential adverse outcome. For patients with malignancy, type and stage of their disease is known to influence life expectancy. We entered the presence of a malignancy as a potential confounder in all multivariable models; however, we did not specifically assess severity of malignant disease in this general surgical population.

Finally, due to the observational character, this study is inherent to unmeasured confounding.

Surgical outcome is influenced by the patients’ preoperative status, severity of disease or surgical procedure and quality of care [26]. In the Netherlands, a high-resource country, accessibility and quality of care are considered equal for all inhabitants. Also, the wide implementation of modern perioperative programmes such as fast-track surgery or goal-directed therapy seems to contribute to a reduced postoperative morbidity. [27, 28].

In conclusion, high-risk vascular surgery and the occurrence of postoperative complications are important prognostic factors of late mortality. Further focus on these groups of patients can contribute to a reduced postoperative morbidity. Improvement in quality of surgical care should be aimed at preventing postoperative complications and thus a better outcome in a general surgical population.

## References

[1] Weiser TG, Regenbogen SE, Thompson KD (2008). An estimation of the global volume of surgery: a modelling strategy based on available data. Lancet.

[2] Khuri SF, Henderson WG, DePalma RG (2005). Determinants of long-term survival after major surgery and the adverse effect of postoperative complications. Ann Surg.

[3] Moonesinghe SR, Harris S, Mythen MG, et al. Survival after postoperative morbidity: a longitudinal observational cohort studydagger Br J Anaesth 2014 10.1093/bja/aeu224 PMC4235571 25012586

[4] Derogar M, Orsini N, Sadr-Azodi O (2012). Influence of major postoperative complications on health-related quality of life among long-term survivors of esophageal cancer surgery. J Clin Oncol.

[5] Pearse RM, Moreno RP, Bauer P (2012). Mortality after surgery in Europe: a 7 day cohort study. Lancet.

[6] Tjeertes EKM, Hoeks SE, Beks SBJC (2015). Obesity—a risk factor for postoperative complications in general surgery?. BMC Anesthesiol.

[7] www.asahq.org 2016

[8] Classification of Surgical Procedures (Document in Dutch) Prismant, Utrecht 2005

[9] Boersma E, Kertai MD, Schouten O (2005). Perioperative cardiovascular mortality in noncardiac surgery: validation of the Lee cardiac risk index. Am J Med.

[10] Dindo D, Muller MK, Weber M (2003). Obesity in general elective surgery Lancet.

[11] cbs.statline.nl, 2014

[12] Noordzij PG, Poldermans D, Schouten O (2010). Postoperative mortality in the Netherlands: a population-based analysis of surgery-specific risk in adults. Anesthesiology.

[13] Al-Omran M, Tu JV, Johnston KW (2003). Outcome of revascularization procedures for peripheral arterial occlusive disease in Ontario between 1991 and 1998: a population-based study. J Vasc Surg.

[14] Cooper GS, Yuan Z, Landefeld CS (1996). Surgery for colorectal cancer: race-related differences in rates and survival among Medicare beneficiaries. Am J Public Health.

[15] Lee DS, Donovan L, Austin PC (2005). Comparison of coding of heart failure and comorbidities in administrative and clinical data for use in outcomes research. Med Care.

[16] Silber JH, Rosenbaum PR, Trudeau ME (2005). Changes in prognosis after the first postoperative complication. Med Care.

[17] Toner A (2013). Hamilton M the long-term effects of postoperative complications. Curr Opin Crit Care.

[18] Pearse RM, Harrison DA, James P (2006). Identification and characterisation of the high-risk surgical population in the United Kingdom. Crit Care.

[19] Chovatiya R (2014). Medzhitov R stress, inflammation, and defense of homeostasis. Mol Cell.

[20] Cardinale F, Chinellato I, Caimmi S (2011). Perioperative period: immunological modifications. Int j of immunopathol and pharmacol.

[21] Vollmar B (2011). [Pathophysiological basis of surgery-linked sepsis] Der Chirurg. Zeitschrift fur alle Gebiete der operativen Medizen.

[22] Kimura F, Shimizu H, Yoshidome H (2010). Immunosuppression following surgical and traumatic injury. Surg Today.

[23] Report of the World Health Organization G International Stitistical Classification of Diseases and Related Health Problems 2004

[24] Merry AH, Boer JM, Schouten LJ (2009). Validity of coronary heart diseases and heart failure based on hospital discharge and mortality data in the Netherlands using the cardiovascular registry maastricht cohort study. Eur J Epidemiol.

[25] Harteloh P, de Bruin K (2010). Kardaun J The reliability of cause-of-death coding in The Netherlands. Eur J Epidemiol.

[26] Bennett-Guerrero E, Hyam JA, Shaefi S (2003). Comparison of P-POSSUM risk-adjusted mortality rates after surgery between patients in the USA and the UK. Br J Surg.

[27] Grocott MP, Martin DS (2012). Mythen MG enhanced recovery pathways as a way to reduce surgical morbidity. Curr Opin Crit Care.

[28] Hamilton MA, Cecconi M (2011). Rhodes A A systematic review and meta-analysis on the use of preemptive hemodynamic intervention to improve postoperative outcomes in moderate and high-risk surgical patients. Anesth Analg.

